# Study on the Mechanical Properties of Crack Mortar Repaired by Enzyme-Induced Calcium Carbonate Precipitation (EICP)

**DOI:** 10.3390/ma17122978

**Published:** 2024-06-18

**Authors:** Gang Li, Deqiang Yan, Jia Liu, Peidong Yang, Jinli Zhang

**Affiliations:** 1 Shaanxi Key Laboratory of Safety and Durability of Concrete Structures, Xijing University, Xi’an 710123, China; t_bag945@126.com (G.L.); 19915445726@163.com (D.Y.); yang_peidong2023@126.com (P.Y.); 2 State Key Laboratory of Coastal and Offshore Engineering, Dalian University of Technology, Dalian 116024, China; jlzhang@dlut.edu.cn

**Keywords:** EICP, mortar, crack, repair method, mechanical properties

## Abstract

As an emerging repair method, the enzyme-induced calcium carbonate precipitation (EICP) technique has the advantages of being highly economical, eco-friendly, and durable. The optimal repair conditions were obtained by taking cement mortar as the research object, adding two types of filling medium, using three EICP-based repair methods to repair the cement mortar with different crack widths, and combining ultrasonic testing and strength testing to evaluate the mechanical properties and repair effects of the repair mortar. The microscopic structure of the mortar was established using mesoscopic and microscopic tests (XRD, SEM, and EDS), thereby revealing the mechanism of repair based on EICP. The test results show that, when quartz sand is used as the repair medium, more calcium carbonate adheres to the cross-section of test samples, and it has a better repair effect. Moreover, the repair effect of the injection method is significantly higher than those of the perfusion and immersion methods, and the ultrasonic wave transit time decreases by 1.22% on average. Based on the combination of quartz sand and EICP repair methods, the calcium carbonate precipitated among the sand granules contributes to a binding effect that strengthens the cohesive force among the sand granules.

## 1. Introduction

With excellent performance, cement-based materials have been widely used in construction, transportation, water conservancy, and other fields. Microcracks appear in cement-based materials over long service periods, due to the coupling effect of loading and environment, resulting in the intrusion of moisture and corrosive ions into the materials. Corrosion failure, expansion failure, and freezing–thawing damage failure consequently occur, shortening the overall structural life cycle [1]. Some scholars have researched the repair methods for material cracks. Ramaglia et al. [2] and Chen et al. [3], respectively, studied the effects of adding silica (SiO_2_) nanoparticles and air entrainers on the mechanical properties of lime mortar. Diaz et al. [4] and Kontić et al. [5], respectively, studied the effects of Textile Reinforced Mortar (TRM) and lime mortar on the mechanical properties of ancient building restoration. All achieved good results. Traditional crack repair methods include surface treatment [6], filling [7], and grouting [8,9]. While the surface treatment method only applies to fine, shallow, watertight, unshrinkable, and inactive cracks, the filling method requires grooves before repair and adds repair materials. It is suitable for cracks wider than 5 mm. The grouting method applies to cracks with a width of less than 2 mm and easily causes secondary pollution. For these reasons, it is extremely important to find an economical, environment-friendly, and durable repair method for easily cracked cement-based materials.

Microbially induced calcite precipitation (MICP) and enzyme-induced calcium carbonate precipitation (EICP) techniques are two new popular research directions in recent years [10,11,12,13,14], both of which utilize urease to hydrolyze urea and introduce a calcium source to generate calcium carbonate into cement soil, thereby improving soil strength and reducing soil permeability. Different from the MICP technique, the EICP technique directly uses urease for urea hydrolysis without involving any biosafety issues. Therefore, it does not need to consider the aerobic environment. Moreover, urease is much smaller in size (12 nm) than bacteria (0.5–3.0 μm), so it is more suitable for the solidification of cohesive fine particle soil, showing broader application prospects. The techniques have been widely applied to foundation stabilization [15], liquefaction treatment [16], rock and earth mass seepage control [17], wind prevention and sand fixation [18], polluted soil repair [13], and other fields. Kidanemariam et al. [19] elaborated on the principle and applicability of the EICP technology and introduced its applications in dust control, self-repairing concrete, and small-volume pavement renovation. Cui et al. [20] adopted a single-phase low-pH EICP treatment to improve soil. The test results show that, compared with traditional two-phase EICP treatment methods, the single-phase low pH method significantly improves the calcium conversion efficiency and the homogeneity of the calcium carbonate dispersion in these sand samples. Zomorodian et al. [21] investigated the resistance to surface erosion of compressed medium-grade silica sand samples treated by different EICP methods. They found that, compared with the untreated sand, all EICP treatment methods significantly increase the erosion resistance of treated sand, and 1.0 M cementing solution is a more feasible option to achieve the optimal erosion resistance. Ahenkorah et al. [22] conducted a direct simple shear (DSS) test and studied the mechanical properties of EICP-treated sand and untreated sand under critical conditions. Based on their study, compared with the treated samples under similar initial conditions, the EICP-treated samples have a higher shear strength, and the final state of the treated sand tends to have a lower percentage of voids than the untreated critical state sand. Rosewitz et al. [23] used trace carbonic anhydrase (CA) to investigate the self-healing mechanism of cement slurry. The crystal growth rate using this method is more efficient, and the healing time is shorter than that of the bacterial method. The research shows that it is an effective mechanism for repairing and strengthening existing concrete structures. Lee et al. [24] studied the properties of the EICP-treated sand specimens using the grouting method and concluded that the EICP-treated sand specimens showed greater softening behavior in the unconfined state. Dong et al. [25] used sucrose and skim milk powder to improve EICP technology to protect the surface of the ancient buildings of Tabia. The results show that sucrose-modified EICP technology can be used as an effective method through which to protect the Tabia site and even ancient buildings. Xu et al. [26] used chitosan combined with EICP technology to treat copper-containing wastewater and found that the addition of calcium sources inhibited the increase in pH and significantly improved the treatment efficiency. Compared with chemical precipitation, ion exchange, and microbiological methods, the EICP technology is low-cost, easy to operate, and environmentally friendly.

The above research indicates that EICP has achieved certain research results in fields such as soil solidification, rock anti-seepage, and cultural relic restoration. However, there is little research on the different methods and mechanisms of EICP repair for mortar cracks. In this study, cement mortar with different crack widths was repaired by taking cement mortar as the research object, adding two types of filling medium (quartz sand and aeolian sand), and using three EICP-based repair methods (immersion, perfusion, and injection), and the mechanical properties and repair effects of repair mortar were evaluated by conducting ultrasonic testing and strength testing. Microscopic and mesoscopic investigations (XRD, SEM, and EDS) were carried out to construct the microscopic structure of the mortar, which revealed the mechanism of EICP repair. All the results of this research have established references and are of great scientific importance to the practical engineering for the repair of cement-based materials using the EICP technique.

## 2. Materials and Methods

### 2.1. Test Materials

PO42.5 common Portland cement (manufactured by Taiyuan Sunnsy Cement Co., Ltd., Taiyuan, China) and ISO standard sand, purchased from Xiamen, were used to prepare mortar samples for the test. Aeolian sand and quartz sand (Nanyang Chufeng Silicon Material Co., Ltd., Nanyang, China) were used as crack-filling materials. The chemical composition of the cement is given in Table 1, and the physical and mechanical properties are given in Table 2. The physical parameters of the sand are given in Table 3. Commercially available soybeans were chosen as the urea source. The soybeans were grown in Heilongjiang Province, with yellow surfaces and full and uniform grains. The test reagents include urea (manufactured by Tianjin Hengxing Chemical Reagent Co., Ltd., Tianjin, China) and calcium chloride (Anhui Yihua Packaging Co., Ltd., Fuyang, China), both of which are analytically pure.

### 2.2. Sample Preparation

In compliance with the standard test method for the stability of cement grout (ISO method), the cement, the standard sand, and the water were blended in a ratio of 1:3:0.5 and evenly stirred using NJ-160A cement mortar mixer (manufactured by Zhejiang Xiyi Testing Machine Manufacturing Co., Ltd., Shaoxing, China). Then, the mortar was vibrated and poured into forms measuring 40 mm × 40 mm × 160 mm. Because cement-based materials are often used with cracks in practical engineering, naturally formed cracks present complicated factors. Therefore, scholars have adopted controlled methods to prefabricate cracks and repair them, including anti-folding prefabrication, cutting prefabrication, and artificial insertion of steel sheets [27]. The steel plate insertion method was used to prepare cracks. Before the final setting of the mortar, three 40 × 40 × 40 mm steel plates were evenly inserted in the middle and both ends of the mortar sample, with a spacing of 40 mm and an insertion depth of about 20 mm. After 24 h of solidification, the mold and iron sheet were removed, and the crack repair work was carried out after 7 days of maintenance.

**Figure 1:**
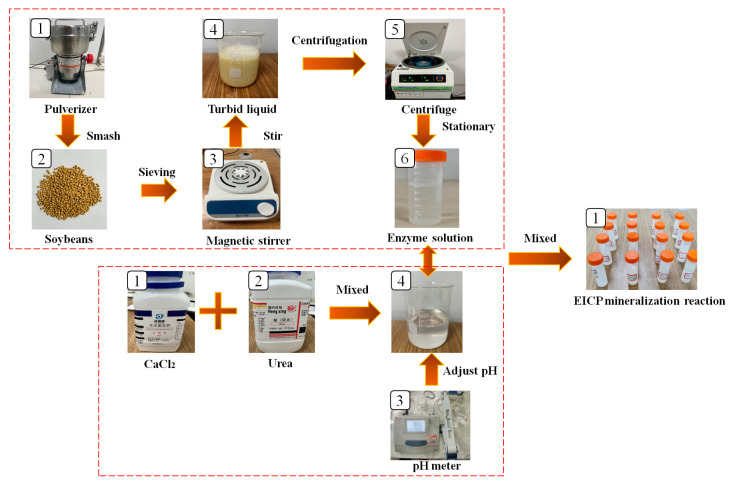
Preparation processes of the enzyme solution and cementing solution.

Fresh soybeans were crushed for 5 to 10 min in an 800A crusher (manufactured by Yongkang Jinwei Electric Appliance Co., Ltd., Yongkang, China) and filtered by a 100-mesh sieve to collect soybean powder. Soybean flour was added to deionized water, stirred by a SN-MS-1 magnetic stirrer (manufactured by Xi’an KTL Instruments Co., Ltd., Xi’an, China) for 1 h, and kept still for 24 h. Then, the solution was placed into a TGL-16G centrifuge (manufactured by Hunan Kecheng Instrument and Equipment Co., Ltd., Changsha, China) and centrifuged for 15 min at 4 °C and 3000 r/min to extract the supernatant solution, and obtain the enzyme solution. Calcium chloride and a urea solution were used to prepare the cementing fluid. The pH values of the two solutions were adjusted with a pH meter, and the crystal form was stabilized by adding skim milk powder. The enzyme solution was mixed with the cementing solution, and the enzyme solution catalyzed the hydrolysis of urea in the cementing solution to obtain carbonate ions and ammonium ions. The calcium ions in the cementing solution reacted with the hydrolyzed carbonate ions to form calcium carbonate precipitates, which were used for repairing mortar cracks. Previous experiments considered the effects of enzyme solution concentration, enzyme solution incorporation ratio, pH value, and the content of skim milk powder on the EICP reaction rate, and obtained the optimal factors, as follows: enzyme solution concentration, 100 g/L; cementing fluid concentration, 1.0 mol/L; skim milk powder content, 6 g/L, pH7.0. See Figure 1 for the preparation processes of the enzyme solution and cementing solution.

### 2.3. Test Methods

**Figure 2:**
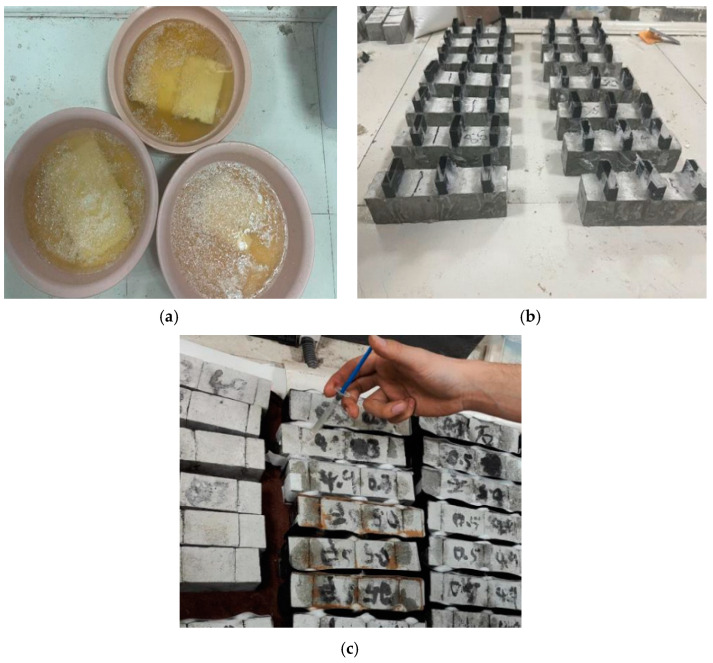
Mortar crack repair methods: (**a**) immersion; (**b**) perfusion;and (**c**) injection.

After the curing of the cement mortar specimen, 60% aeolian sand and quartz sand were added into the crack as repair media. To determine the repair method suitable for cracked mortar, 3 EICP repair methods, including immersion, perfusion, and injection, were used in the test to repair the cracked mortar samples (see Figure 2). As shown in the figure, the cement solution was first prepared, at a concentration of 1.0 mol/L, for the immersion method, and then the same volume of enzyme solution, with a concentration of 100 g/L, was added and fully mixed. The mortar sample was immersed into the solution, with a pH value of 7.0, and repaired for 10 days at 20 °C. After that, the mortar sample was taken out of the solution and kept still for 7 days, until the sample surface was fully hardened. For the perfusion method, 3 mL enzyme solution, with a concentration of 100 g/L, was added into the mortar sample through the self-made perfusion tank, and then 3 mL of the cement solution, with a concentration of 1.0 mol/L, was added. The above perfusion process was repeated every 8 h. The mortar sample was repaired for 10 days at 20°C in the solution, with a pH value of 7.0. After that, the mortar sample was taken out of the solution and kept still for 7 days, until the sample surface was fully hardened. For the injection method, a volume of enzyme, with a concentration of 100 g/L, and the same volume of cementing solution were concentrated at 1.0 mol/L into a beaker, and the sample was allowed to react for 30 min in the solution, with a pH of 7.0 at 20 °C. The reaction liquid was extracted and injected into cracks until they were filled with the reaction liquid. Absorbent balls were placed at two ends of each crack to absorb the excess reaction liquid. The reaction liquid was injected once every 8 h during the test, and this process continued for 10 days. Then, the mortar sample was kept still indoors for 7 days, until the sample surface was fully hardened.

**Figure 3:**
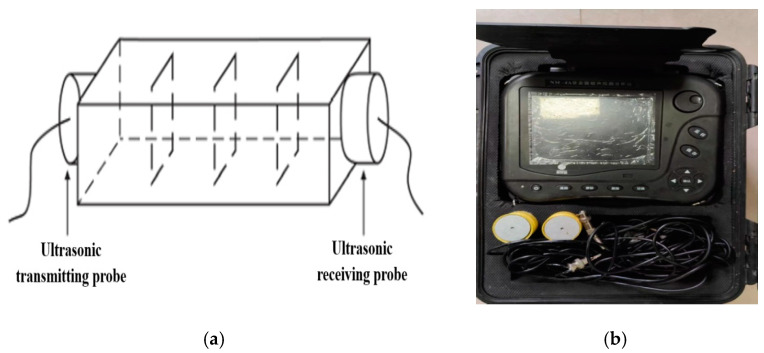
Testing of ultrasonic wave transit time: (**a**) schematic drawing and (**b**) physical image.

**Figure 4:**
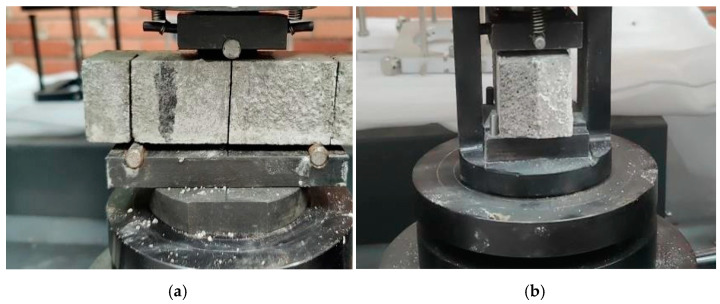
Testing of bending strength and compressive strength: (**a**) bending strength and (**b**) compressive strength.

After the completion of mortar sample curing, ultrasonic testing, bending strength testing, and compressive strength testing were carried out using an NM-4A non-metal ultrasonic detector (made by Beijing Koncrete Engineering Testing Technology Co., Ltd., Beijing, China) and CMT5605 pressure testing machine (manufactured by Shenzhen Sansi Testing Instrument Co., Ltd., Shenzhen, China). The effects of the repair method, repair medium, and crack width were taken into account during the test. The experimental plan is shown in Table 4, and the experimental equipment is shown in Figure 3 and Figure 4.

## 3. Results and Analysis

### 3.1. Analysis of Ultrasonic Testing Results of the Mortar Sample

**Figure 5:**
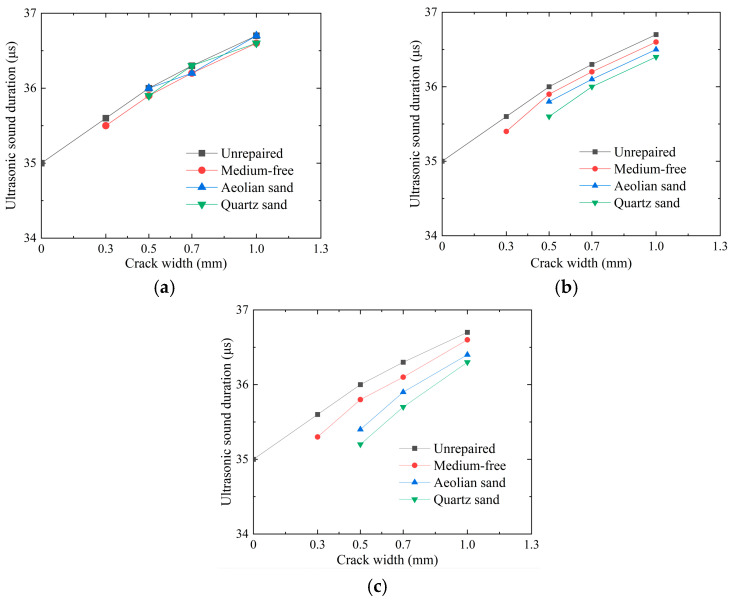
Curve of ultrasonic wave transit time of cement samples under 3 EICP repair methods versus crack width: (**a**) immersion; (**b**) perfusion;and (**c**) injection.

In a typical case, the larger the sample crack width, the longer the acoustic emission distance. Therefore, ultrasonic testing is of great significance for evaluating repair effects on cracked mortar. Figure 5 presents the curves of mortar ultrasonic wave transmission time under the immersion, perfusion, and injection methods versus crack width. As can be seen from the figure, there is a time lag for the transmission of the peak diffusion energy of the unrepaired sample to the receiver. Without the repair medium, these repair methods do not vary greatly in terms of ultrasonic wave transit time. After the addition of a repair medium, the ultrasonic wave transmission time of the sample under the immersion method does not decrease significantly. This may be because the reaction liquid in the immersion method reaches the crack through diffusion, and when it reaches the crack surface, a large amount of calcium carbonate precipitation causes congestion, and it is difficult for the liquid to reach the crack interior. The immersion method requires a lot of enzyme solution and cementing fluid, which are costly and difficult to apply to large components in real-world engineering. However, the ultrasonic wave transmission time of the sample under the perfusion method decreased by 0.37% on average, and that of the sample under the injection method decreased by 0.98% on average, which is in good agreement with the conclusion of reference [28]. This may be because the perfusion method allows direct injection into cracks, so the perfusion method can act on cracks more accurately. The ultrasonic wave transmission time of the injection method is reduced the most because it can reach the crack directly through the syringe, and it is not easy to produce a surface Doucet phenomenon. The injection method uses the least amount of repair solution and is completely feasible for engineering applications. When the injection method is applied to the repair of mortar cracks, and aeolian sand is added as the repair medium, the ultrasonic wave transmission time decreases by 0.76% on average. When quartz sand is added, the ultrasonic wave transmission time decreases by 1.22% on average. The main reason for the above situation is that, when the perfusion and injection methods are used, and quartz sand is used as the repair medium, a better EICP solidification effect is produced than that with aeolian sand because the cracks are filled with quartz sand more compactly, resulting in a significant decrease in ultrasonic wave transmission time.

### 3.2. Analysis of Mechanical Property Results of the Mortar Sample

**Figure 6:**
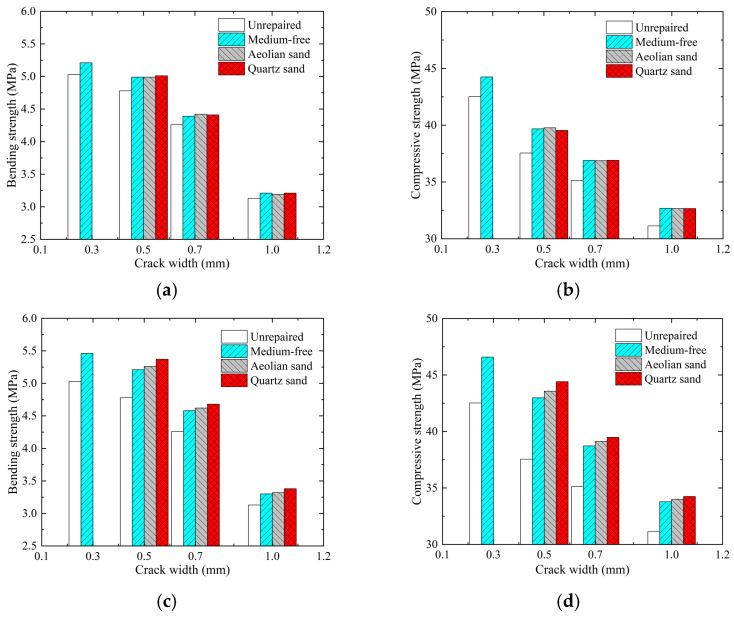

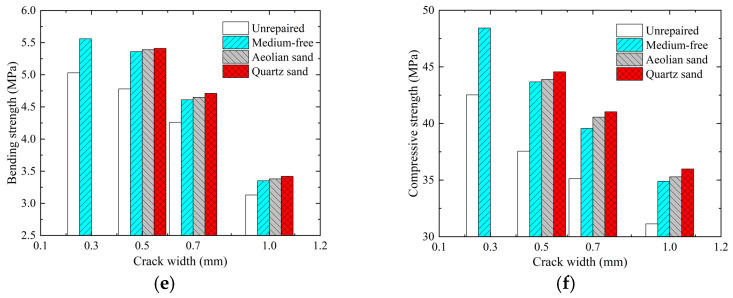
Histogram of sample strengths under 3 EICP repair methods versus crack widths: (**a**) bending strength for immersion method; (**b**) compressive strength for immersion method; (**c**) bending strength for perfusion method; (**d**) compressive strength for perfusion method; (**e**) bending strength for injection method; and (**f**) compressive strength for injection method.

Both sample bending strength and compressive strength can reflect the repair effect of cracked mortar. Figure 6 presents the histogram of sample bending and compressive strengths under the immersion, perfusion, and injection methods versus crack widths. As shown in the figure, the bending and compressive strengths of mortar samples show overall trends of decreasing gradually with increasing crack width. This trend suggests that the increase in crack width has a considerable effect on the general properties of mortar tests, which is in good agreement with the conclusion of reference [29]. Compared with unrepaired samples, the mortar samples repaired by EICP experienced a significant increase in both bending strength and compressive strength. Under the immersion method, the strength increase rate of the mortar sample is the lowest, and the maximum bending strength and compressive strength of the mortar sample are only 5.2 MPa and 44.5 MPa at a crack width of 0.3 mm. Moreover, the strength of a mortar sample filled with a repair medium is similar to that of a sample without a repair medium. Compared with the immersion method, the specimens filled with repair media under the perfusion method showed an increase of 10.04% to 12.34% in bending strength, 16.03% to 18.27% in compressive strength, and 2.00% to 4.21% in combined compressive and bending strength. Under the injection method, both the bending and compressive strengths increased to their maxima after repair, in which the maximum bending strength was 5.5 MPa and the maximum compressive strength was 48.5 MPa. When the crack width is 0.5 mm under the injection method, and quartz sand is used as the repair medium, the maximum increase in sample compressive strength reaches 13.90%, increased by 1.92 times compared to the compressive strength of the specimen treated by the perfusion method, and by 3.65 times compared to the compressive strength of the specimen treated by the immersion method. From this perspective, when the injection method is applied, and quartz sand is used as the repair medium, the mortar sample has higher bending and compressive strengths, providing a better repair effect and coinciding with the results of ultrasonic testing. The main reason for the above situation is that, when the immersion method works, a mineralization reaction occurs between the enzyme solution and cementing solution, and the generated CaCO_3_ crystals fail to cement the repair medium and the mortar sample into a whole. As a result, the sample filled with the repair medium has a similar strength to the sample not filled with the repair medium. Under the action of the perfusion and injection methods, the addition of a repair medium has a significant impact on improving the sample strength, and the injection method is more effective than the perfusion method in repairing fine cracks. During repair using the injection method, an enzyme and cementing solution can be transferred into deep cracks to realize mineralization reactions and ensure a better cementing effect between the repair medium and mortar sample. Moreover, the overall repair effect will not be degraded by surface clogging.

### 3.3. Analysis of Calcium Carbonate Uptake Rate of Mortar Samples

**Figure 7:**
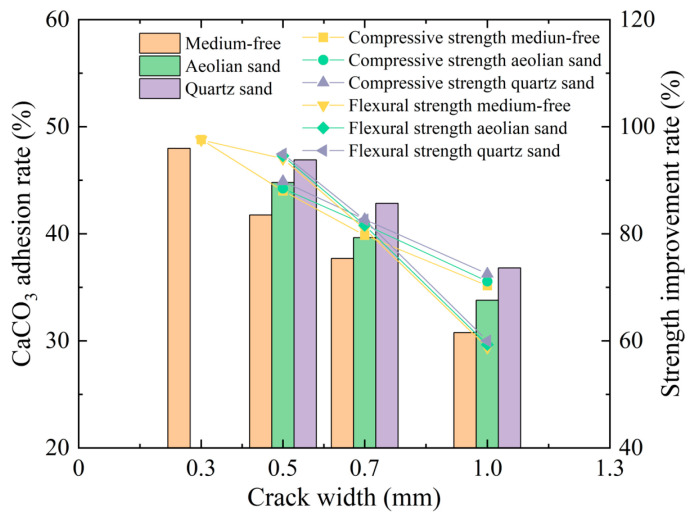
Histogram of calcium carbonate uptake rate and strength increase under the action of the injection method versus crack width.

The CaCO_3_ uptake rate and strength increase rate during repair using EICP can directly reflect the repair effect of cracked mortar. The calcium carbonate on the cross section of mortar was measured by means of the software Image J, and the calcium carbonate uptake rate was calculated. Figure 7 presents the histogram of mortar sample calcium carbonate uptake rate and strength increase rate under the action of the injection method versus crack width. As the figure shows, both the flexural and compressive strengths of the mortar samples gradually decrease as the crack width increases. After repair through the injection method, the calcium carbonate uptake rate of the mortar sample increases significantly and reaches its maximum when quartz sand is used as the repair medium. The strength increase rate is within the range of 87.48% to 95.36% for a sample with a crack width of 0.5 mm, 79.30% to 82.23% for a 0.7 mm crack width sample, and 67.22% to 71.86% for a 1.0 mm crack width sample. The strength of the mortar sample with quartz sand as its repair medium is substantially higher than that of the mortar sample with aeolian sand as its repair medium. The fundamental reasons for thisare that quartz sand has a larger particle size, and the pores between the sand granules are also enlarged to facilitate the penetration of the enzyme and cement solution. However, aeolian sand has fine and small granules and low permeability, so it is complicated for the enzyme and cementing solution to fully penetrate into the sample and realize a mineralization reaction. As a result, the overall cementing effect is degraded, and the strength of mortar samples decreases. It is concluded that, when quartz sand is used as the repair medium, the mortar sample has a higher strength and better repair effect, which is in good agreement with the conclusion of the previous analysis.

### 3.4. Analysis of Apparent Repair Effect of the Mortar Sample

**Figure 8:**
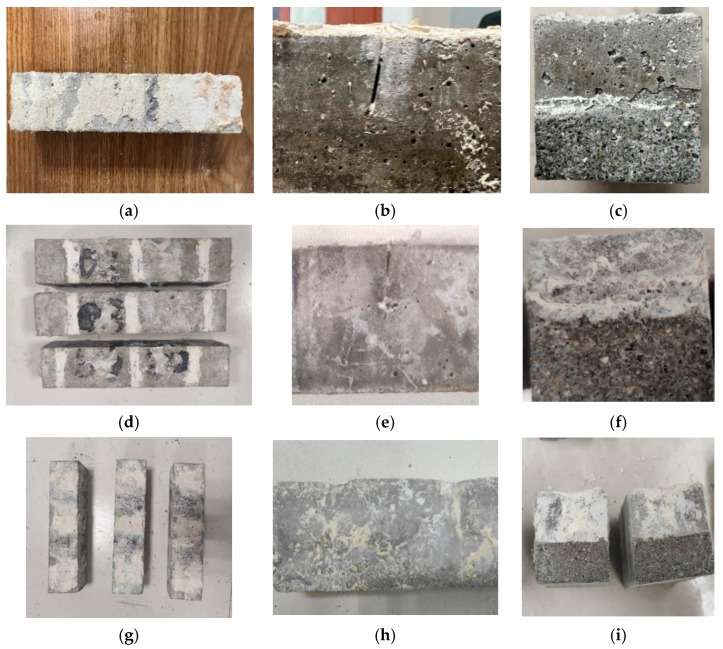
The apparent repair effect of mortar samples under the three repair methods: (**a**) immersion method on the surface; (**b**) immersion method from the side; (**c**) immersion method in cross section; (**d**) perfusion method on the surface; (**e**) perfusion method from the side; (**f**) perfusion method in cross section; (**g**) injection method on the surface; (**h**) injection method from the side; and (**i**) injection method in cross section.

Apparent repair effect is one of the most direct methods through which to assess the impact of mortar crack repair through EICP. Figure 8 shows the surface, side, and cross-section repair effects of mortar samples under the three studied repair methods. As can be seen from the figure, the calcium carbonate generated by the immersion method adheres to the surface of the mortar sample and does not reach the interior of the crack. the perfusion method-restored mortar samples not only had calcium carbonate attached in the perfusion tank, but also a small amount of calcium carbonate that reached the inside of the crack. This is consistent with the results of ultrasonic detection. The sample repaired by the injection method has a flat surface, without any obvious sign of cracking. All lateral cracks of the sample are filled with a repair medium. A large amount of a white crystal substance can be seen at the cross-section. The white crystal substance comprises calcium carbonate crystals, produced by mineralizing the EICP. Calcium carbonate crystals are formed by rapidly injecting an enzyme and cementing solution into cracks, and then the repair medium and mortar are cemented into a whole to repair cracks. With the injection method, an enzyme and cementing solution can be injected deep into cracks. The resulting calcium carbonate crystals can effectively cement the repair medium into a whole, thus filling the pores of cracks. With the progress of the mineralization reaction, calcium carbonate crystals solidify the repair medium from the inside out, and the repair medium is cemented into a whole with the mortar at each crack to improve the overall mechanical properties of mortar samples.

### 3.5. Analysis of the Mechanism of Action for the Repair of Mortar Samplesthrough EICP

**Figure 9:**
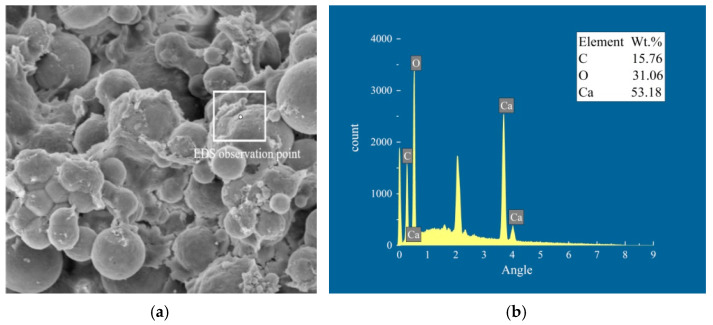
Morphology and EDS spectrum of the materials in the mortar sample cracks under the action of the injection method: (**a**) EDS station and (**b**) EDS spectrum of the materials.

**Figure 10:**
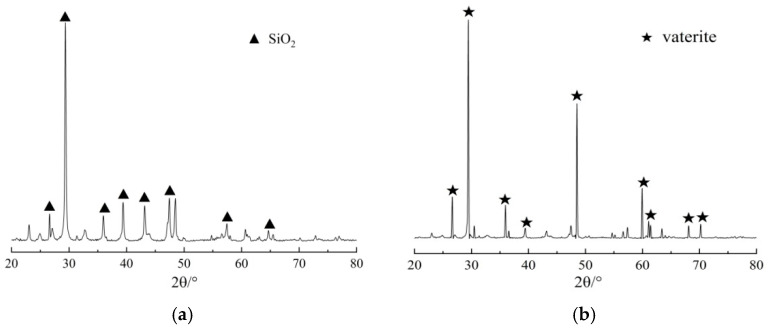
XRD spectrum of the mortar sample under the action of the injection method: (**a**) unrepaired and (**b**) repaired.

To reveal the mechanism of action for the repair of mortar samples through EICP, the diffraction spectrum, microstructure, and mineral composition of the mortar sample were observed by carrying out XRD, SEM, and EDS tests. Figure 9 presents the EDS spectrum of the mortar sample repaired under the action of the injection method, and Figure 10 presents the XRD spectrum of the mortar sample under the action of the injection method. As can be seen from the figures, the minerals generated by EICP take the shape of round balls, and the contents of C, O, and Ca are 15.76%, 31.06%, and 53.18%, respectively, indicating that the resulting CaCO_3_ crystals are vaterite. According to the XRD spectrum, the unrepaired mortar sample mainly contains SiO_2_, while the repaired mortar sample mainly contains vaterite, with main peak 2*θ* values of 29.37° and 29.24°, respectively. The results suggest that the CaCO_3_ crystals generated by the mineralization of EICP have the effect of a cementing repair medium.

**Figure 11:**
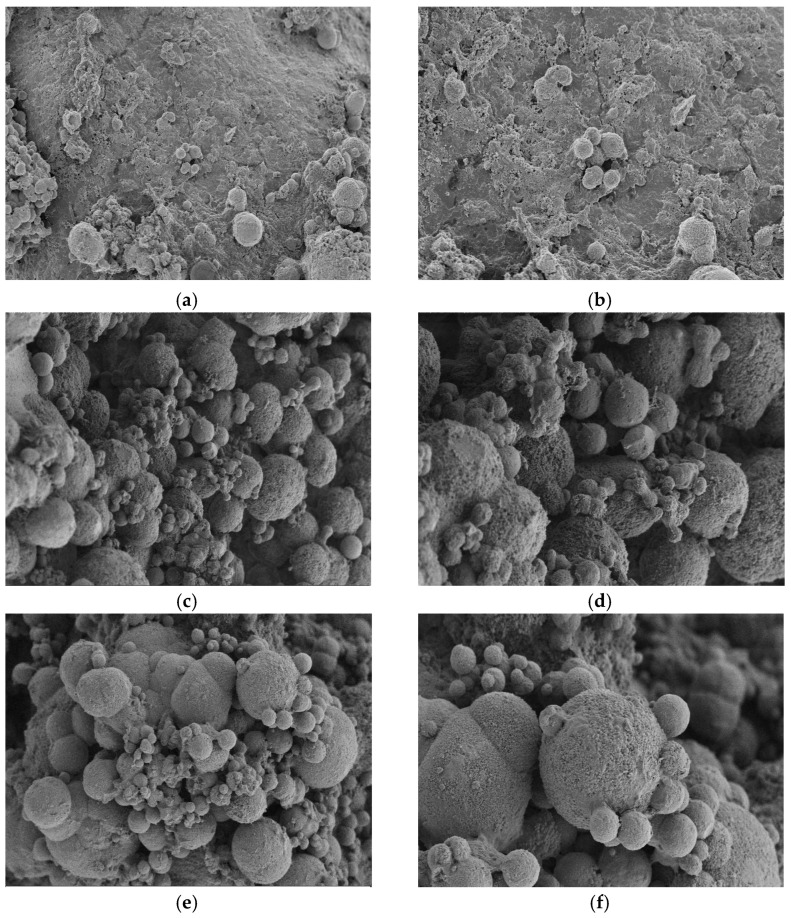
Microstructure of mortar test block under different repair methods: (**a**) 1000× magnification for immersion; (**b**) 2000× magnification for immersion; (**c**) 1000× magnification for perfusion; (**d**) 2000× magnification for perfusion; (**e**) 1000× magnification for injection; and (**f**) 2000× magnification for injection.

**Figure 12:**
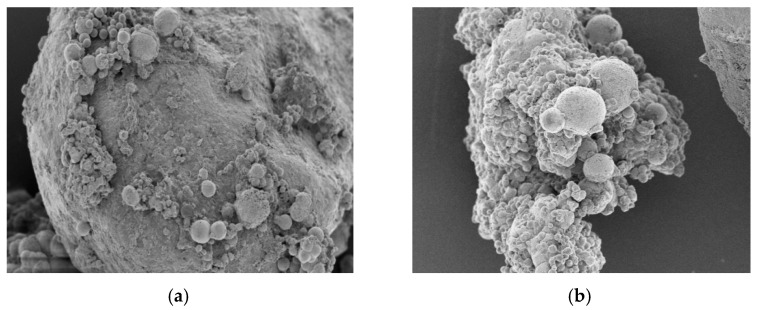
Comparison of different repair media with the injection method: (**a**) aeolian sand and (**b**) quartz sand.

Figure 11 shows the microstructures of mortar test blocks under three different EICP restoration methods, Figure 12 shows the microstructure of different repair media added by the injection method. As can be seen from the figure, the CaCO_3_ crystals generated under the three EICP restoration methods are mostly spherical and mainly play the roles of covering, filling, and cementing [30]. It is concluded from the previous analysis that the resulting round minerals are vaterite. The number of CaCO_3_ crystals generated by the immersion method is small, and the crystals attached to the crack surface are also small, so it is difficult to achieve a cementation effect in the crack. A large amount of vaterite was produced under the perfusion and injection methods, which evenly covered the crack surface of the cement mortar. The crystal shape generated under the perfusion method is an irregular sphere, such as a stickball or ellipsoid. The sample repaired using the injection method contains more CaCO_3_ crystals, which take the shape of full round balls and have higher crystal mobility and dispersity to become absorbed and evenly distributed on the surface of cracks more effectively. This is in line with the conclusions of the previous study. When aeolian sand is used as the repair medium, small numbers of spherical calcium carbonate crystals can be seen on the surface of the sand particles, which may be because the surface of the fan sand particles is smooth, and it is difficult to attach more calcium carbonate crystals. When using quartz sand as the repair medium, a large number of calcium carbonate crystals can be clearly seen attached. The surface of the crystals is rough, and small spherical particles adhere to the large particles, accelerating the bonding process inside the cracks. The combination of quartz sand and EICP repair methods ensures a closer contact between quartz sand and mortar sample and generates more cluster crystals through cementation. The calcium carbonate precipitating between sand grains contributes a bonding effect, which improves the cohesive force between sand grains. Then, the cracks are gradually filled and slowly cemented into a whole to repair cracked mortar samples.

## 4. Conclusions

In this study, the optimal repair conditions were obtained by taking cement mortar as the research object, adding two types of filling medium (quartz sand and aeolian sand), using three EICP-based repair methods (immersion, perfusion, and injection) to repair the cement mortar with different crack widths, and combining ultrasonic testing and strength testing to evaluate the mechanical properties and repair effects of the repair mortar. The mechanism of action for repair through EICP was revealed by conducting mesoscopic and microscopic testing (XRD, SEM, and EDS) and observing the diffraction spectra, microstructures, and mineral compositions of mortar samples. The main conclusions are as follows:

1. The unrepaired sample has a larger ultrasonic wave transmission time. However, the time decreases significantly after repair with the filling medium. When the perfusion and injection methods are applied, and quartz sand is used as the repair medium, the EICP technique has a better solidification effect, and the ultrasonic wave transmission time decreases by 1.22% on average, under the action of the injection method with quartz sand as the filling medium.
2. Compared with unrepaired samples, the mortar samples repaired by EICP experienced a significant increase in both bending strength and compressive strength. Under the immersion method, the sample filled with the repair medium has a similar strength to that of the sample not filled with the repair medium. When the injection method is applied, the maximum compressive strength increase rate of the sample with quartz sand as the repair medium reaches 13.90%, 1.92 times greater than that with the perfusion method, and 3.65 times greater than that with the immersion method. From this perspective, when the injection method is applied and quartz sand is selected as the repair medium, the test sample of repair mortar has higher bending and compressive strengths, thus contributing to a better repair effect.
3. After repair through the injection method, the test sample has a flat surface, and all lateral cracks are filled with a repair medium. There is a large number of white crystals at the cross-section, which are calcium carbonate crystals produced by the mineralization of EICP. Calcium carbonate crystals are formed by rapidly injecting an enzyme and cementing solution into cracks, and then the repair medium and mortar are cemented into a whole to improve the overall mechanical properties of mortar samples.
4. The minerals generated by EICP take the shape of round balls and mainly contain vaterite. The main peak 2*θ* values are 29.37° and 29.24°, suggesting that there is a large number of CaCO_3_ crystals, and these crystals take the shape of full round balls. Based on the combination of quartz sand and EICP repair methods, the calcium carbonate precipitating between sand grains contributes a bonding effect, which improves the cohesive force between sand grains. Then, the cracks are gradually filled and slowly cemented into a whole to repair mortar cracks.
5. At present, the EICP repair method is mainly applicable to cement mortar. In the future, EICP technology has great potential to repair the cracks in lime-based mortar, concrete, and reinforced concrete. According to the strength and durability testing of the repaired samples, the EICP repair mechanism can be further revealed, coupled with numerical simulation and microscopic testing, in future research.

## Figures and Tables

**Table 1:** Mineral composition of the cement.

Cement	Na_2_O	MgO	Al_2_O_3_	SiO_2_	SO_3_	Cl^-^	CaO	Fe_2_O_3_
*ω*%	2.73	3.00	8.26	24.99	2.20	0.04	51.42	4.03

**Table 2:** Physical and mechanical properties of the cement.

Compressive Strength (MPa)		Bending Strength (MPa)		Setting Time (min)		Specific Surface Area (m^2^/kg)	Stability
7 d	28 d	3 d	28 d	Initial condensation	Final condensation	358.0	Qualified
27.2	50.8	5.5	8.6	172.0	234.0		

**Table 3:** Basic physical indicators of sand.

Sand Type	*G*_S_	*ρ*_dmax_	*ρ*_dmin_	*d*_10_	*d*_30_	*d*_60_	*C*_u_	*C*_c_
Standard sand	2.652	1.730	1.460	0.140	0.500	0.900	6.428	1.984
Aeolian sand	1.850	1.720	1.470	0.086	0.130	0.215	2.500	0.914
Quartz sand	2.660	1.820	1.460	0.080	0.100	0.144	1.800	0.868

**Table 4:** Test scheme of mortar crack repair through EICP.

Repair Method	MediumType	Crack Width (mm)	Time (d)
Immersion	None	0, 0.3, 0.5, 0.7, 1.0	10
	Aeolian sand	0, 0.5, 0.7, 1.0	10
	Quartz sand	0, 0.5, 0.7, 1.0	10
Perfusion	None	0, 0.3, 0.5, 0.7, 1.0	10
	Aeolian sand	0, 0.5, 0.7, 1.0	10
	Quartz sand	0, 0.5, 0.7, 1.0	10
Injection	None	0, 0.3, 0.5, 0.7, 1.0	10
	Aeolian sand	0, 0.5, 0.7, 1.0	10
	Quartz sand	0, 0.5, 0.7, 1.0	10

## Data Availability

The data are not publicly available for privacy reasons. The data presented in this study are available from the corresponding author.
